# Terahertz Spectroscopy for Proximal Soil Sensing: An Approach to Particle Size Analysis

**DOI:** 10.3390/s17102387

**Published:** 2017-10-19

**Authors:** Volker Dworak, Benjamin Mahns, Jörn Selbeck, Robin Gebbers, Cornelia Weltzien

**Affiliations:** Department Engineering for Crop Production, Leibniz-Institute for Agricultural Engineering and Bioeconomy (ATB), Max-Eyth-Allee 100, 14469 Potsdam, Germany; bmahns@atb-potsdam.de (B.M.); jselbeck@atb-potsdam.de (J.S.); rgebbers@atb-potsdam.de (R.G.); cweltzien@atb-potsdam.de (C.W.)

**Keywords:** THz, transmission measurement, proximal soil sensing, soil absorption, soil parameters, precision agriculture

## Abstract

Spatially resolved soil parameters are some of the most important pieces of information for precision agriculture. These parameters, especially the particle size distribution (texture), are costly to measure by conventional laboratory methods, and thus, in situ assessment has become the focus of a new discipline called proximal soil sensing. Terahertz (THz) radiation is a promising method for nondestructive in situ measurements. The THz frequency range from 258 gigahertz (GHz) to 350 GHz provides a good compromise between soil penetration and the interaction of the electromagnetic waves with soil compounds. In particular, soil physical parameters influence THz measurements. This paper presents investigations of the spectral transmission signals from samples of different particle size fractions relevant for soil characterization. The sample thickness ranged from 5 to 17 mm. The transmission of THz waves was affected by the main mineral particle fractions, sand, silt and clay. The resulting signal changes systematically according to particle sizes larger than half the wavelength. It can be concluded that THz spectroscopic measurements provide information about soil texture and penetrate samples with thicknesses in the cm range.

## 1. Introduction

Precision agriculture contributes to solving local and global problems in agricultural production by taking into account the within-field variability of soil and crops. The adoption of agricultural measures to site-specific requirements saves resources, minimizes the negative impacts on the environment and reduces costs by conserving gasoline and chemicals such as fertilizers and pesticides [1,2,3]. Manufacturers of agricultural machinery have designed machines that can react to spatial soil variability at the field scale. Fertilizer spreaders for site-specific application were first on the market, and high-resolution maps are currently needed to support the meter-range variability [1,4,5]. These maps are expensive and time consuming to generate by conventional soil sampling and laboratory analysis, especially for soil parameters, which change over time [6]. Therefore, in situ proximal soil sensing is an important research focus [7,8,9] that aims to develop on-the-go, nondestructive or minimal-impact sensors to measure the local soil parameters. Some sensors, such as pH sensors [10,11] and penetration sensors [12] directly measure the parameter of interest; others, such as conductivity sensors [13,14], eddy current sensors [15,16], and visible to near-infrared (Vis-NIR) spectrometers [17,18,19], require calibration; and some sensors only indirectly measure the parameters and require additional modeling [20] and calibration with other sensor outputs.

Terahertz (THz) spectroscopy is a new promising technique for proximal soil sensing. The first investigations of soils by THz radiation [21] were carried out with fixed frequencies. They demonstrated that lower frequencies of approximately 300 gigahertz (GHz) can penetrate soil samples to depths of over centimeters, resulting in the acquisition of bulk information instead of the two-dimensional (2D) surface information obtained from light-based sensors such as Vis-NIR spectrometers. Thus, the frequency range around 300 GHz is regarded as a good compromise between the penetration of and interaction (scattering, absorption, and reflection) with the sample [22]. The first results obtained at three frequencies (340, 351, 360 GHz) demonstrated the imaging possibility of the THz setup and that there are frequency-dependent variations in the signal amplitudes [22]. To analyze the spectral behavior with many more wavebands, we established a new THz setup with backward wave oscillator (BWO) tubes. These BWOs are able to sweep the frequency and produce high output power in the milliwatt range. BWOs are preferred over laser-based setups [23,24], Gunn elements [25], or upcoming quantum cascade lasers [26]. These emit less power than BWOs for continuous wave (CW) output, and thus BWOs yield a better signal-to-noise ratio. Our new setup features high sensitivity, high spectral resolution, and high reproducibility and is therefore most suitable to analyze soil samples. To the best of our knowledge, the frequency range from 250 GHz to 370 GHz has rarely been used for soil analysis. In particular, THz spectra in this range have not been related to the chemical composition of soils (“fingerprinting”) and only a few attempts have been made to inspect physical properties [21]. Thus, we expect new results that may even contradict current theoretical assumptions, e.g., a flat response over the frequency. Soil, which typically contains a complex mixture of inorganic and organic materials with different physical, chemical and biological properties, is a very difficult sample to analyze. In our research, we first try to discriminate the effects of different soil properties on THz. By studying the effects of soil components individually, we hope to establish detailed—maybe even causal—response models which might not be possible if the effects of many soil properties are considered simultaneously. As a first stage of our research agenda we focus on the mineral particle size distribution (particle size fractions), which is an important soil parameter because it is related to the surface area (ion exchange capacity), pore size distribution and related hydraulic properties, as well as to soil organic matter (clay–humus complexes), and thus dominates the soil behavior [27]. The following questions are considered:

- How does the particle size influence the spectral behavior, and is there a limit or saturation of the signal amplitudes?
- How does the sand fraction, with a large particle size, interfere with the THz radiation (≈wavelength (λ))?
- How does the sample thickness influence the spectral behavior?
- Which fraction dominates the signal content of mixed fractions?
- For the soil fractions, silt and clay, which are relevant for the adsorption of plant nutrients, high penetration by THz radiation is expected, because of the low Rayleigh scattering by fractions with particle sizes much smaller than the wavelength (<<λ). Thus the question is, do soils with similar textures differ in THz transmission?

These questions will be answered experimentally. The high penetration depth and screening possibility of the THz spectroscopy setup will be shown, illustrating its promising application to proximal soil sensing.

## 2. Materials and Methods

The methods section is separated into three subsections describing (a) the samples and their preparation with respect to particle size fractions, (b) the measurement setup and its performance, and (c) the theoretical expectations about THz scattering and diffraction.

### 2.1. Soil Samples

To analyze particle size effects on THz damping, contrasting materials with respect to particle size were used. These include natural soil materials (quartz sand and Illite clay) and artificial material (glass spheres). The natural soil materials were preconditioned (e.g., fairly standardized and homogeneous) in order to reduce the inherent complexity of natural soils, which could have imposed additional difficulties on these initial experiments. To validate the particle size related effects on THz transmission that were observed with the preconditioned soil materials, we used artificial, highly standardized material (glass spheres). The quartz sand samples were from construction sand, bought from Hornbach Baumark AG (Bornheim, Germany), with particle sizes ranging from 0.1–0.3 mm, 0.3–0.6 mm and 0.8–1.6 mm. The loss on ignition was <0.5% (according to DIN EN 12620 for construction sand). The quartz sand was further sieved in our lab to fractions of 1.4–1.25 mm, 1.25–1 mm, 1–0.63 mm, 0.63–0.5 mm, 0.5–0.4 mm, 0.4–0.3 mm, 0.3–0.2 mm, 0.2–0.1 mm, 0.1–0.063 mm, 63–45 µm and 45–0 µm. The Illite clay sample was Green Clay™ from Laboratoires Argiletz (Lizy-Sur-Ourcq, France) and contained particles <2 µm of Illite clay. The glass spheres were obtained from Kugel Pompel (Vienna, Austria) in fractions of 2.9–2.4 mm, 2.3–2 mm, 1.85–1.55 mm, 1.3–1 mm,1–0.75 mm, 0.6–0.4 mm, 0.25–0.15 mm, 0.2–0.1 mm and <50 µm. The glass spheres of different sizes were used to confirm the findings from the natural soil samples. Soda-lime glass should have higher purity (less chemical components) than the natural samples, should show high absorption in the THz region similar to the IR range, and thus should be easily detectable.

The description of mineral particle size distribution in soil science is based on the analysis of samples that are passed through a 2 mm screen [28]. The particles <2 mm are classified into three main fractions (clay, silt, sand) as given in Table 1.

The particle sizes and THz wavelengths/frequencies correspond as shown in Table 1. This is relevant for discussing the interaction between particle size and THz radiation. To evaluate the instrument capability to identify finer particle fractions, the quartz sand was sieved to further fractions: 1.4–1.25 mm, 1.25–1 mm, 1–0.63 mm, 0.63–0.5 mm, 0.5–0.4 mm, 0.4–0.3 mm, 0.3–0.2 mm, 0.2–0.1 mm, 0.1–0.063 mm, 63–45 µm and 45–0 µm. These fractions of quartz and clay were compared to samples of glass spheres (2.9–2.4 mm, 2.3–2 mm, 1.85–1.55 mm, 1.3–1 mm, 1–0.75 mm, 0.6–0.4 mm, 0.25–0.15 mm, 0.2–0.1 mm and <50 µm).

In natural soils, different particle size fractions often show different chemical compositions. Even though we did not expect strong influences of chemical composition on electromagnetic waves in the THz range covered by our spectrometer, we analyzed the elemental composition of our samples with an X-ray fluorescence spectrometer (Niton XL3t Ultra 955, Thermo Scientific, Billerica, MA, USA). The main chemical components are listed in Table 2 (elements with atomic numbers less than that of magnesium (12) could not be measured due to the detection limits).

Table 2 reveals some significant differences in the chemistry of the small and large fractions. In the small fractions (<63 µm), K and Al were clearly enriched. However, the differences within the small (<63 µm) and the large fractions (>400 µm) were small.

Accordingly, the chemical composition of the glass spheres was analyzed as shown in Table 3.

Differences in the chemical composition of the different glass sphere fractions were not significant. The low Fe and Ti contents listed in Table 3 illustrate that the spheres were made of pure soda-lime glass. The CaO content should be approximately 10%; Na_2_O (14%) was not detected.

### 2.2. THz Setup

**Figure 1:**
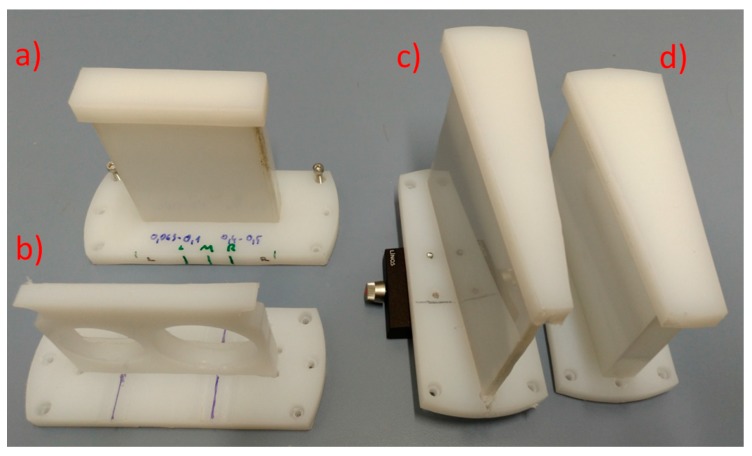
High-density polyethylene (HDPE) sample holders: (**a**) rectangular shape; (**b**) dish holder; and (**c**,**d**) wedge shape.

For THz spectroscopy, all samples were measured in sample holders made of high-density polyethylene (HDPE) with 2 mm wall thickness. HDPE was used because of its excellent transmittance in the 300 GHz range. The sample holders had rectangular or wedge shapes (Figure 1).

The sample holders in Figure 1 have 80 mm widths and 60 mm heights. The rectangular holder is 10 mm thick, and the sample thickness in the wedge shapes ranges from 0 to 20 mm or from 10 mm to 30 mm.

**Figure 2:**
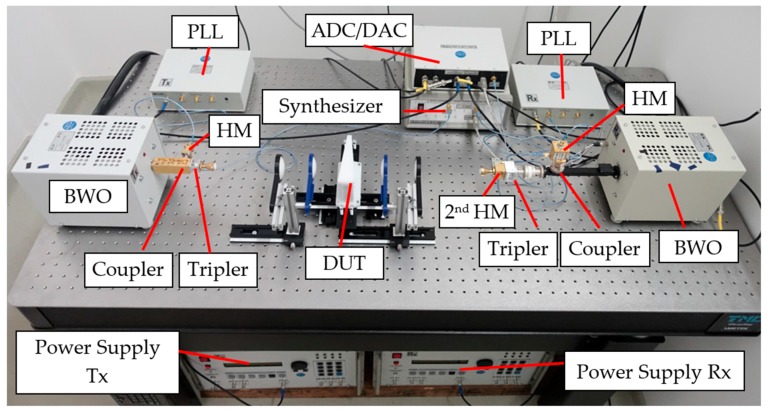
Photo of the THz spectrometer setup: PLL, phase locked loop; HM, harmonic mixer; Tx, transmitting channel; Rx, receiving channel; ADC, analog-to-digital converter; DAC, digital-to-analog converter; and DUT, device under test.

**Figure 3:**
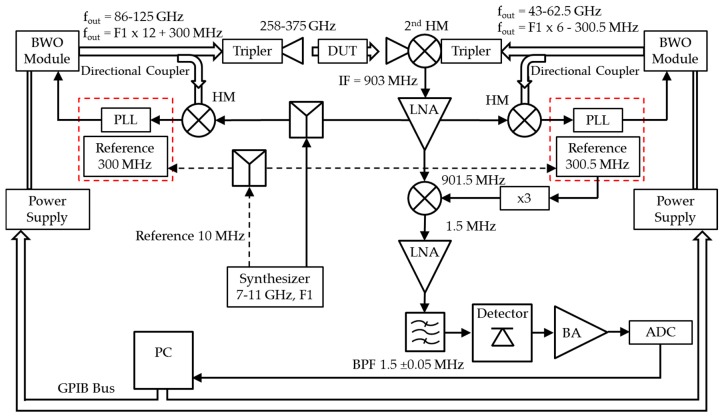
Schematic of the THz spectrometer.

The THz spectrometer is constructed with BWOs. The output power of a BWO is coupled through a frequency tripler, and the output of the tripler is transmitted through the sample or lens system via a horn antenna. The transmitted signal is detected by a second harmonic mixer (HM; Virginia Diodes, Inc., Charlottesville, VA, USA), which is the most sensitive detection method because of the low noise floor of the detector (<10–15 NEP). The disadvantage of the second HM is that an additional BWO and tripler are needed to drive the reference input of the mixer with a synchronous frequency input (Figure 2 and Figure 3). In our case, a synchronous frequency offset enabled the generation of an AC-coupled output signal without 1/f noise. This setup measures from 258 GHz to 375 GHz, and we used 0.03 GHz increments for the spectral resolution.

The setup is mounted on an optical table to enable fast rearrangements of different optical setups. A schematic of the setup of the spectrometer from ELVA 1, St. Petersburg, Russia, is shown in Figure 3.

**Figure 4:**
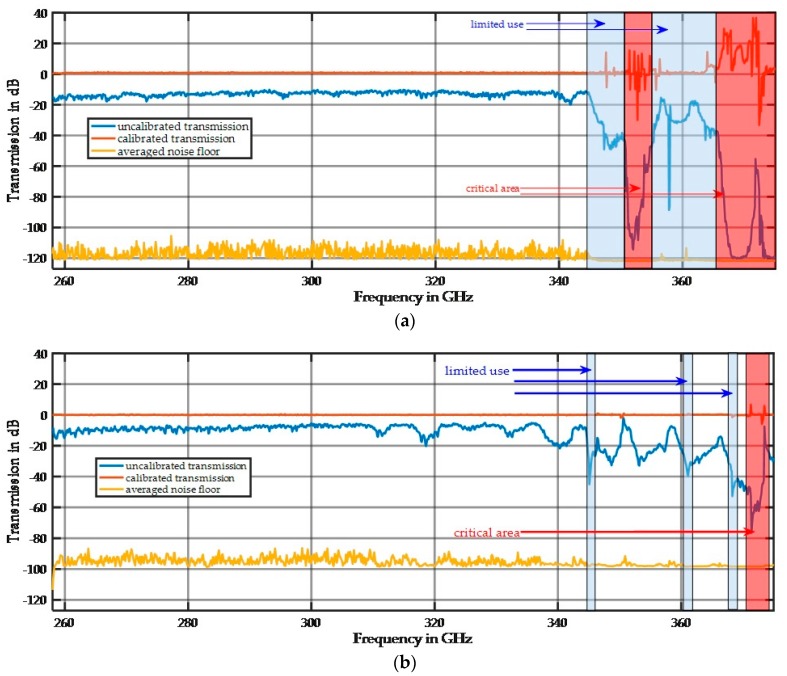
(**a**) Plot showing the spectral behavior of setup 1 without a sample. The blue regions indicate frequency ranges with limitations; these ranges can only be used with adequate signal amplitudes. The red regions have highly restricted use; (**b**) Plot showing the spectral behavior of setup 2 without a sample.

The overall performance including the optical setup with four lenses is demonstrated in Figure 4. The limitations of the waveguide frequency matching performance cause some areas in the spectrum to experience strong signal reductions. Two setups were used for the measurements:

- Setup 1: Rx-tube 1 with a second HM WR3.4SHM and ADC-preamp 1.
- Setup 2: Rx-tube 2 with a second HM WR2.8SHM and ADC-preamp 2.

The difference between the noise floor and the system signal, including the lens setup, over the frequency range from 258 GHz to 345 GHz is typically 96 dB for setup 1 and 80 dB for setup 2. The calibrated signal line in Figure 4 demonstrates high reproducibility because there is no visible difference over multiple scans. The high-frequency performance in Figure 4b can be enhanced by 10 dB by removing the Faraday isolator between the second HM and coupler.

### 2.3. Scattering and Diffraction

Given the focus on the particle size of soil samples, chemical (e.g., molecular composition) effects are not addressed in this research. In what follows, particle refers to a strong material of mineral composition. Since our samples are almost free of organic matter only the theory of interaction of an electromagnetic wave with an accumulation of strong nonmetallic particles will be presented. Given this boundary condition we will review the theory about the interferences of electromagnetic waves with solid material of specific size regarding several physical effects.

#### 2.3.1. Abbe’s Resolution Limit

The resolution limit describes the inability to distinguish two particles separated by distance *l* if the wavelength (*λ*) of the light is not sufficiently short and the numerical aperture (NA = *n·sin*(*α*)) of the objective is not sufficiently high. This is commonly known as the “Abbe’s resolution limit”.

(1) $$l=\frac{\lambda }{2\cdot n\cdot sin\left(\alpha \right)}$$

The NA of the setup is ½; therefore, for particle sizes and particle distances smaller than *λ*, the measured signals are not dependent on the individual particle. For larger particle sizes, each particle generates its own diffraction. The number of particles in the sample holder could be very high, depending on the thickness of the holder. Therefore, small particles should react the same way as a homogenous sample does, and large particles should cause multiple diffractions and thereby scattering.

#### 2.3.2. Rayleigh Scattering

For particles smaller than the wavelength, the Rayleigh equation [29] describes the scattering behavior.

(2) $$I=I_{0}\frac{1}{R^{2}}\frac{1+cos^{2}\left(\theta \right)}{2}{\left(\frac{2\pi }{\lambda }\right)}^{4}{\left(\frac{n^{2}-1}{n^{2}+2}\right)}^{2}{\left(\frac{d}{2}\right)}^{6}$$

The parameter *R* stands for the distance between the scattering particle and the viewer/detector. The angle *θ* is between the incoming direction and the viewing direction, and *λ* stands for the wavelength. The refraction index is given by *n*, and the particle diameter is given by *d*, whereby the particle size interferes with the scattering intensity at a power of six, resulting in small particles that produce small scattering and signal damping caused via absorption. Therefore, low signal damping is expected for silt and clay.

#### 2.3.3. Mie Scattering

Mie scattering describes the scattering behavior of particles (spheres) in the dimension of the wavelength. Mie mostly calculated the behavior of gold spheres [30], but his universal solution has been applied to non-conducting spheres [31].

(3) $$\left(\frac{E_{∥s}}{E_{⊥s}}\right)=\frac{ie^{-ikr}}{r}\left(\begin{array}{cc} S_{2} & 0\\ 0 & S_{1} \end{array}\right)\left(\begin{array}{c} E_{∥e}\\ E_{⊥e} \end{array}\right)$$

The Mie scattering amplitudes for both directions are as follows:

(4) $$S_{1}\left(\vartheta \right)=\sum_{n=1}^{\infty }\frac{2n+1}{n\left(n+1\right)}\left\{a_{n}\pi_{n}\left(\cos\vartheta \right)+b_{n}\tau_{n}\left(\cos\vartheta \right)\right\}\;\mathrm{and}$$

(5) $$S_{2}\left(\vartheta \right)=\sum_{n=1}^{\infty }\frac{2n+1}{n\left(n+1\right)}\left\{a_{n}\tau_{n}\left(\cos\vartheta \right)+b_{n}\pi_{n}\left(\cos\vartheta \right)\right\},$$

where *π_n_* and *τ_n_* are Mie angular functions,

(6) $$\pi_{n}\left(cos\vartheta \right)=\frac{1}{\sin\left(\vartheta \right)}P_{n}\left(cos\vartheta \right)\;\mathrm{and}$$

(7) $$\tau_{n}\left(cos\vartheta \right)=\frac{d}{d\vartheta }P_{n}\left(cos\vartheta \right),$$

where *P_n_* is the associated Legendre polynomial and *a_n_* and *b_n_* are the Mie coefficients.

**Figure 5:**
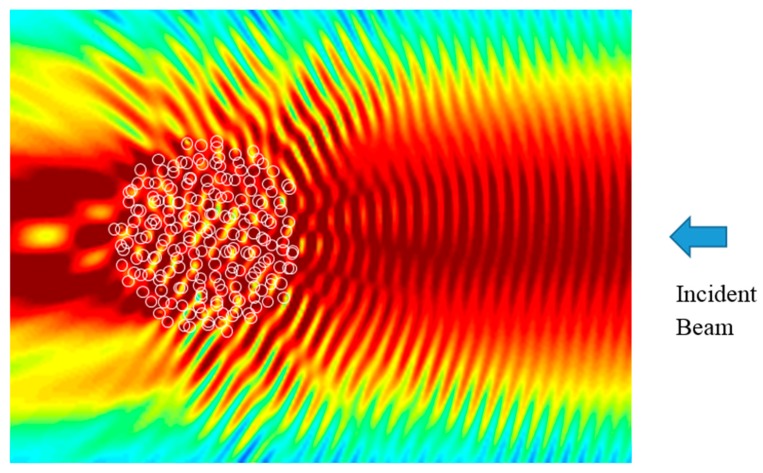
Scattering behavior of 500 arbitrarily arranged spheres. The color gradient shows the local intensity. Simulation result of *Celes* software [32] (Karlsruher Institut fürTechnologie, Karlsruhe, Germany). The incident beam originates from the right side.

With respect to the soil sample behavior, a multiparticle solution is a more adequate model for a centimeter-thick soil sample. *Celes* software [32] (Karlsruher Institut fürTechnologie, Karlsruhe, Germany) for the Matlab environment enables scattering simulation of thousands of spheres in a randomly arranged cluster. Figure 5 shows the complex distribution of a scattered electromagnetic wave. This distribution changes with the random arrangement of the particles, and the transmitted beam has more or less energy depending on the number of maxima falling in the optical path of the beam.

**Figure 6:**
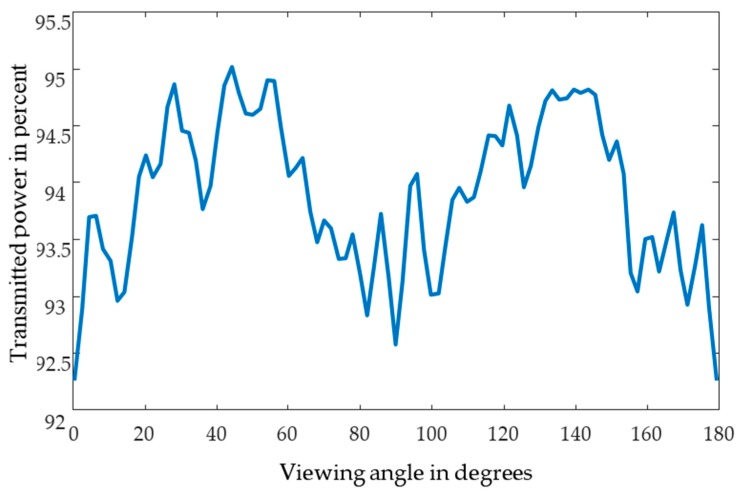
Plot of the *Celes*-simulated transmitted intensity at different viewing angles of an arbitrary cluster of 500 spheres.

Complex scattering behavior exists for perfect spheres of the same diameter. The arbitrary arrangement and the variation in the sample holder could be represented by a rotation of the cluster. Figure 6 shows the transmitted intensity depending on the viewing angle.

**Figure 7:**
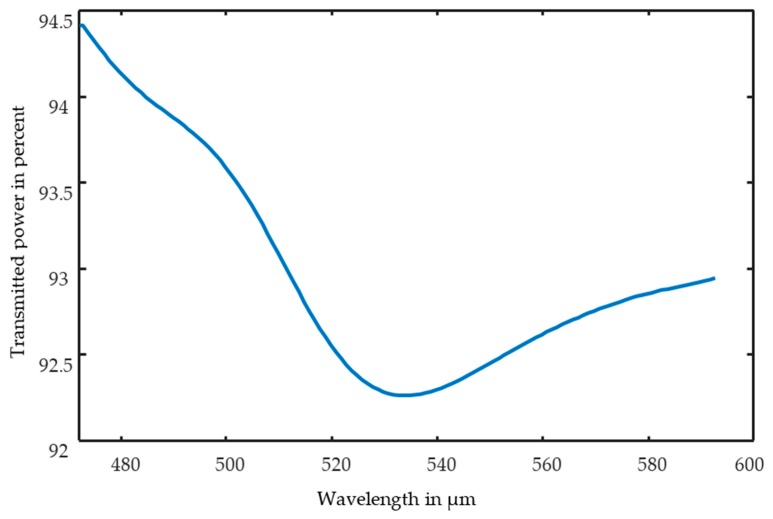
Wavelength dependency of the transmitted power for a cluster.

The plot in Figure 6 demonstrates that the transmitted intensity highly depends on the geometric arrangement of the spheres. Therefore, a high dependence on the local measurement point is expected for this measurement. Additionally, the transmitted intensity changes with the wavelength, as shown in Figure 7.

The authors expect similar or more complex scattering of randomly shaped particles in a random distribution of particle sizes from, e.g., 300 µm to 400 µm, which represents one part of the distribution of the sieved soil samples.

## 3. Results

According to the objectives, the first part of our investigations addressed the particle size dependency and the related spectral behavior of the THz transmission based on samples of (relatively) homogeneous particle fractions. In the second part, mixed fractions were assessed with respect to their averaged damping behavior. Third, the penetration ability for silt and clay was analyzed. This third characteristic concerns a fundamental question in soil analysis with THz radiation, since a volume measurement is addressed.

### 3.1. Particle Size Dependency

The particle size distribution covers a range from less than 2 µm to 2 mm (see Table 1), in which the sand fraction falls in the range of Mie scattering. The spectral behavior of the different particle sizes will be demonstrated first, the average damping of the different particle sizes and the sample thickness dependency second, and the particular effects at the half-wavelength third.

#### 3.1.1. Spectral Behavior

**Figure 8:**
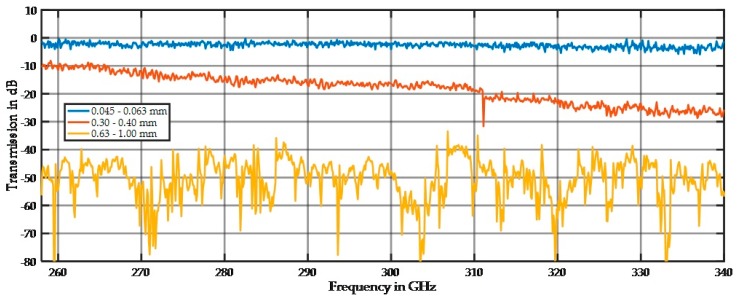
Spectral distribution of three quartz particle size fractions at 11 mm sample thickness.

**Figure 9:**
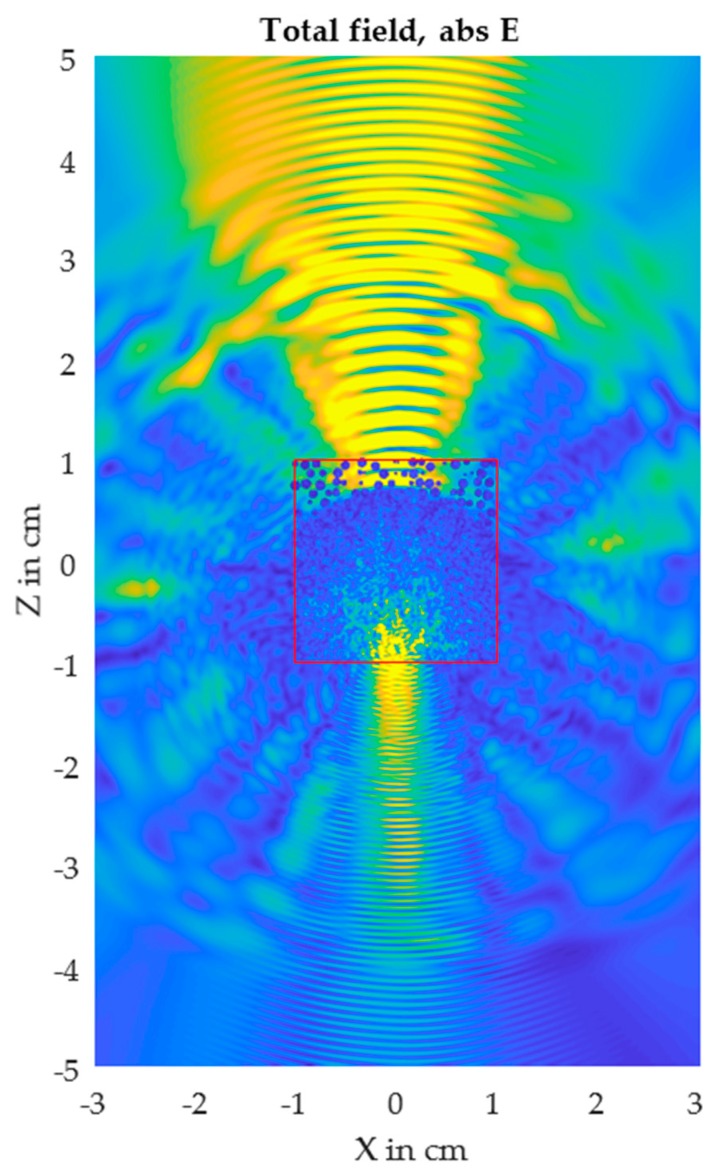
Simulated scattering behavior of 6345 particles, 1 mm in diameter, at a wavelength of 1 mm. A 20 mm-thick sample was simulated. The incident beam originates from the top axis.

**Figure 10:**
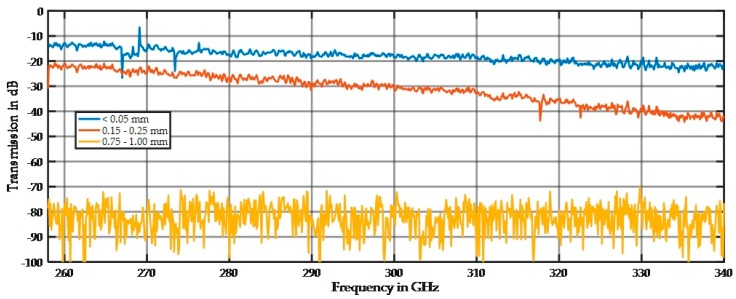
Spectral distribution of three glass particle size fractions at 11 mm sample thickness.

The influence of the particle size on the spectral behavior is demonstrated in Figure 8, Figure 9 and Figure 10. Figure 8 shows the spectra for the 45–63 µm, 300–400 µm, and 630–1000 µm fractions. All spectra are far away from the noise floor, and the prominent spiking up and down is reproducible as long as the sample position remains unchanged. After changing the sample position, the minima and maxima moved to slightly different frequencies, but the overall shape of the spectrum remains the same. The prominent spikes in the spectrum of the large fraction are extremely sharp. We assume that this is a result of resonant scattering in terms of multiple oriented scattering out of the main beam or highly distributed scattering in all directions.

Soda-lime glass is known for poor optical performance (high absorption) in the infrared region, and this was also true for the THz region (Figure 10).

The 45–63 µm fraction is in the Rayleigh scattering range with a small damping of approximately 3 dB and a small increasing damping effect at higher frequencies. For the 0.63–1.0 mm particles, the damping of −50 dB is high, and strong spikes are visible. Figure 9 shows the simulated scattering at a 1 mm wavelength of a 20 mm-thick sample of large spheres 1 mm in diameter. The main beam is reflected and just a small amount can transmit through the sample, and only a small amount of intensities of multiple scattered waves are visible on the other side. This low transmittance causes the high damping of the spectrum, and the number of spikes depends on the number of maxima that fall in the detection beam path.

Figure 10 shows that the damping of approximately 19 dB by glass is significantly higher than that by quartz. For small particles, the Rayleigh scattering is less dominant; therefore, a higher portion of absorption exists for glass. In the Mie scattering regime, the amplitudes of the minimum and maximum values for glass are reduced compared to those for quartz, but the overall damping is also higher than that for quartz.

#### 3.1.2. Damping Behavior

**Figure 11:**
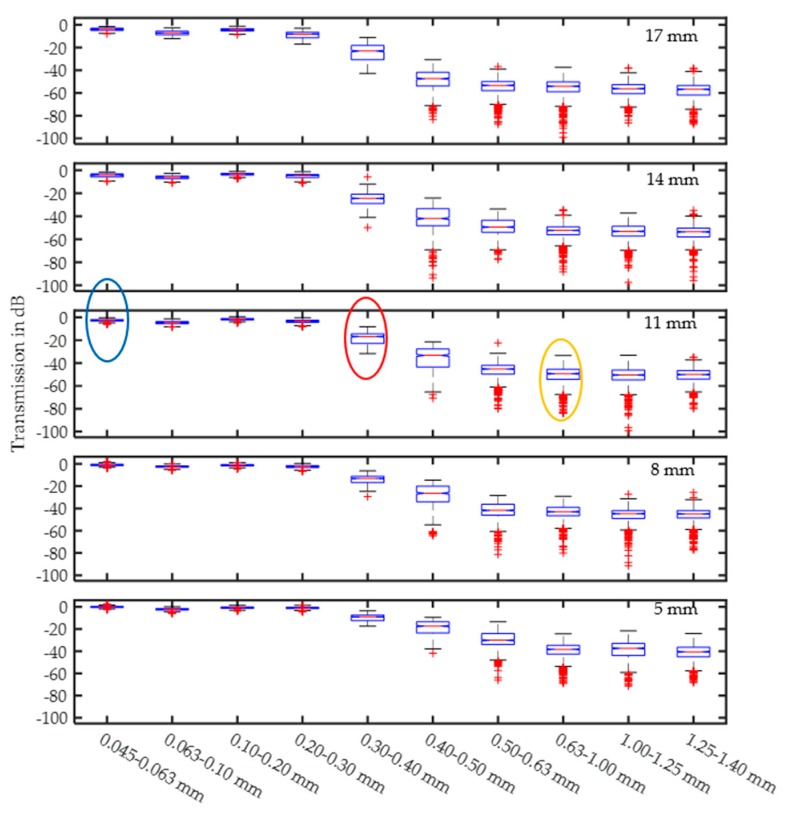
Averaged signal amplitudes for different quartz fractions at different sample thicknesses.

Figure 11 shows the behavior for all fractions at different sample thicknesses. All data in each spectrum were averaged across the full frequency range and plotted. Colored ellipses correspond to the examples in Figure 10.

Figure 11 demonstrates that the damping increases as soon as the particle size reaches the range of half the wavelength. In the range of the wavelength, the damping saturates at a high level of approximately −60 dB for a 17 mm sample thickness. For particle sizes smaller than or equal to one-fourth of the wavelength, the damping saturates at small values. For all thicknesses, the 0.063–0.100 mm fraction has slightly higher damping. With respect to Table 2, the additives cannot be the reason for this damping because the values are decreased compared to those in the smaller fractions. Additionally, the sample thickness has a linear influence on the damping, while the shape and tendencies are similar. The number of outliers (red +) represents the extreme resonant scattering frequencies.

**Figure 12:**
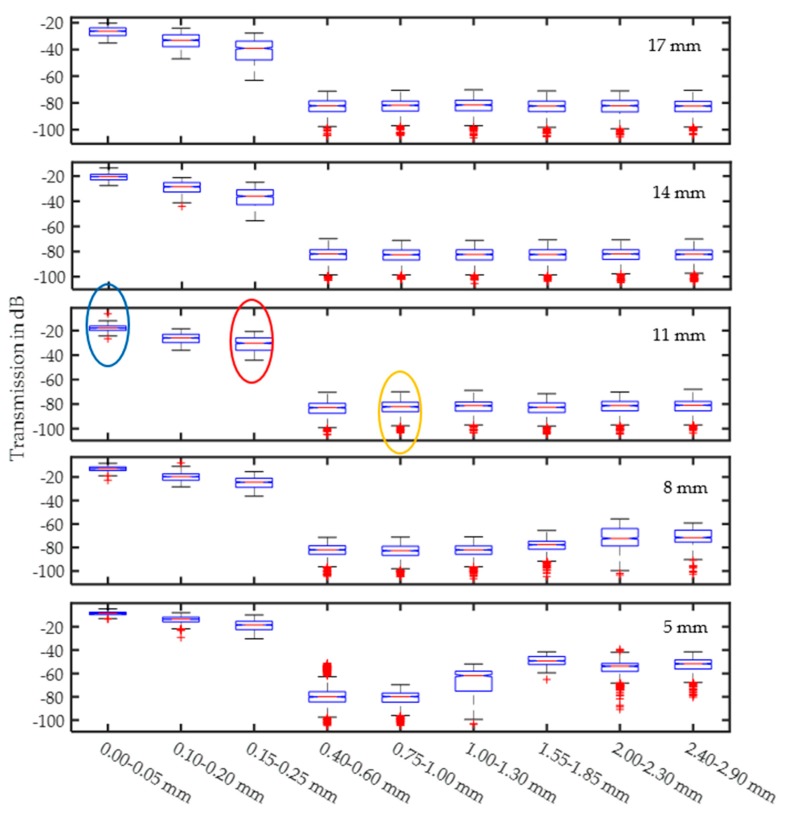
Averaged signal amplitudes for different glass fractions at different sample thicknesses.

Figure 12 shows that the behavior of the glass fractions is similar to that of quartz. The main difference is the high damping level, which is caused by the higher absorption of soda-lime glass. For small sample thicknesses and large particle sizes, the wedge sample holder is not 100% fillable due to geometric constraints. This causes the reduced damping for large particles because of the greater amount of air in the measurement beam in place of the particles that do not fit. Thicker samples show the typical behavior, and saturation exists even for very large particles.

#### 3.1.3. Particular Effects at the Half-Wavelength

**Figure 13:**
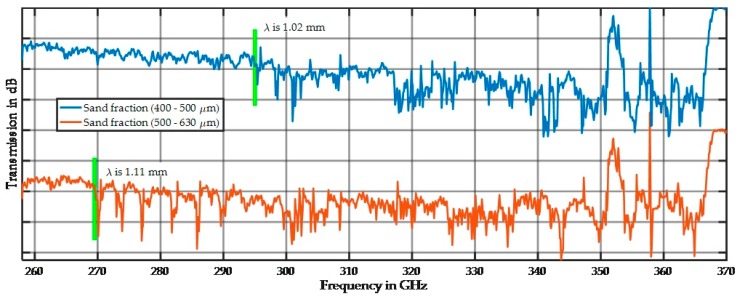
Spectral behavior of two sand fractions in the 10 mm sample holder. Stronger spiking begins around the half-wavelength (green line).

In this subsection, the results for particle sizes approximately half the wavelength are shown. Figure 13 shows the spectral response of two quartz fractions with an offset of −80 dB to separate the spectra.

**Figure 14:**
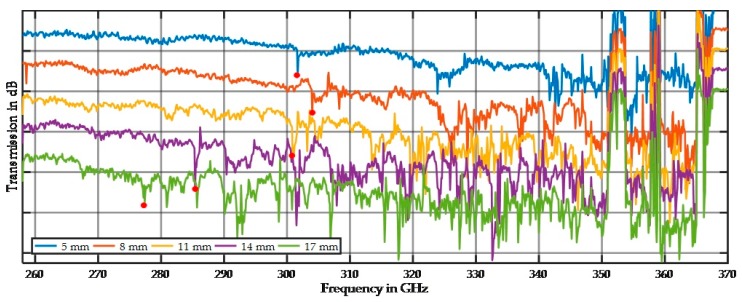
Spectral behavior of five different sample thicknesses for the fraction from 400 µm to 500 µm. The red dots indicate the first spike in each spectrum. For better visibility, an offset of −10 dB was applied between each spectrum.

Both spectra show relatively small variations at lower frequencies up to the half-wavelength, at which point spiking starts. Multiple scattering appears to be more likely for a combination of matching particle arrangement and wavelength. Figure 14 demonstrates this effect depending on the sample thickness and therefore the number of particles that can cause resonant scattering.

The first spike (indicated by the red dot in Figure 14) shifts to lower frequencies for thicker samples. The statistical chance of finding a stochastic arrangement for resonant scattering appears to depend on the number of particles. This spiking behavior was reproduced at different sample positions, but the effect was not observed at all positions, which is why we call it statistical chance. For a given sample thickness the spiking indicates a particle size fraction, but it will take multiple measurements at different sample positions. Therefore, we will establish a mechanical scanner to measure a full sample region (“imaging”).

### 3.2. Mixed Fractions

**Figure 15:**
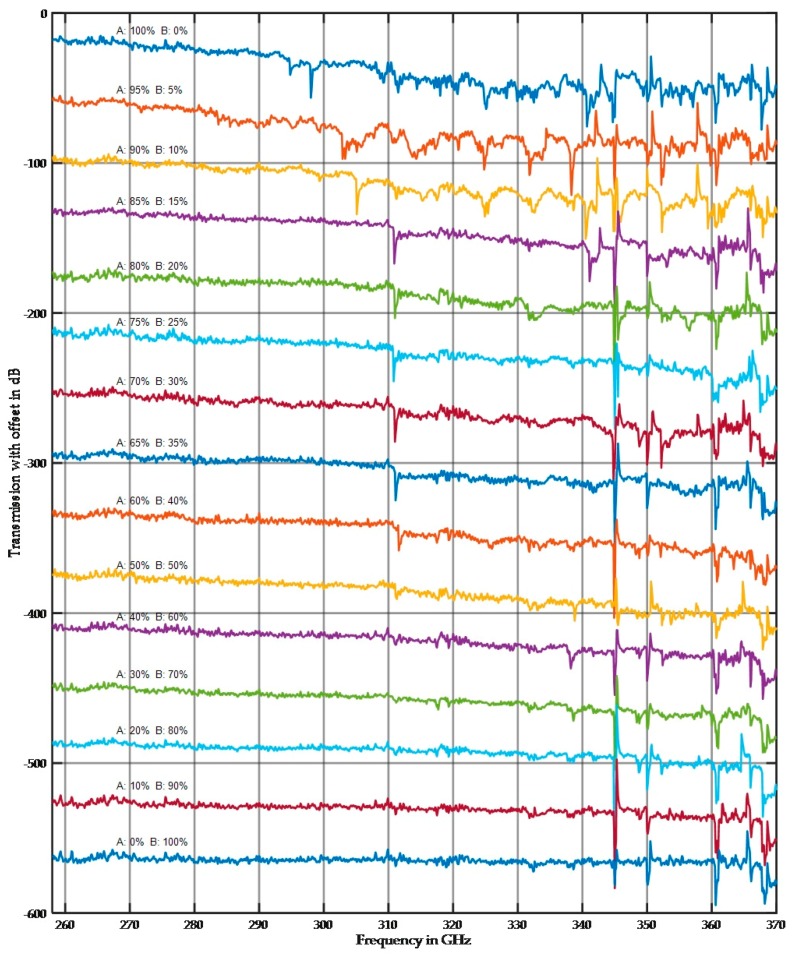
Measurement of mixed fractions. Fraction A is a 0.4–0.5 mm fraction, and fraction B is a 63–100 µm fraction. The percent composition is indicated in the figure. Selected spectra were plotted.

**Figure 16:**
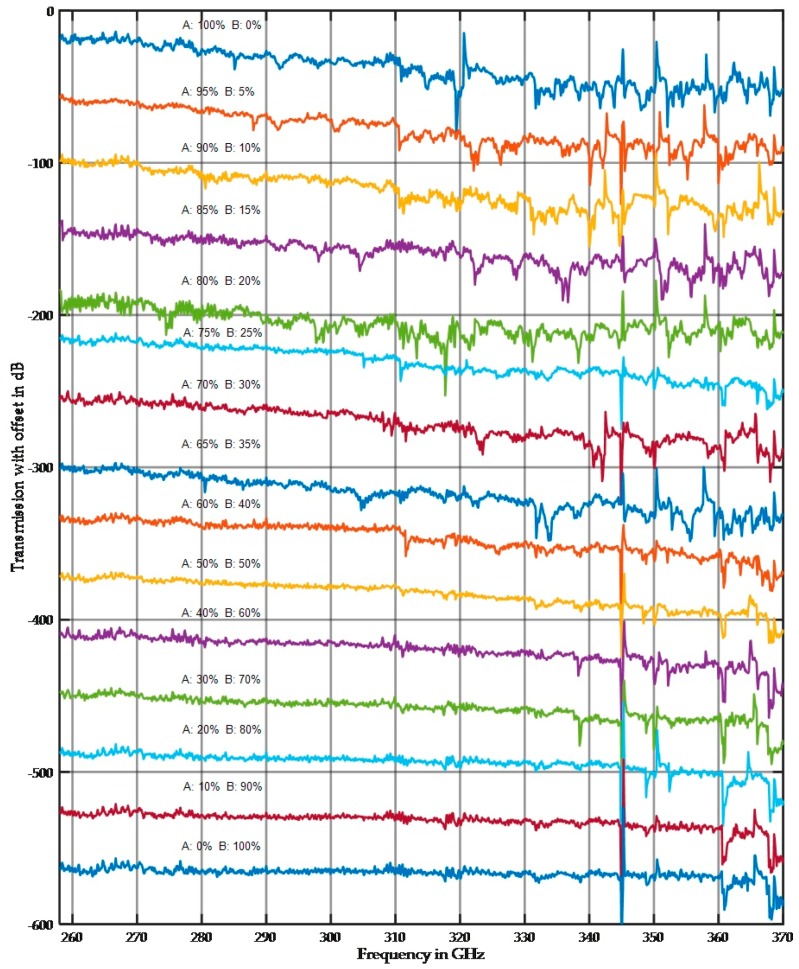
Measurement of the same mixed fractions at different positions. Fraction A is a 0.4–0.5 mm fraction, and fraction B is a 63–100 µm fraction. The percent composition is indicated in the figure. Selected spectra were plotted.

The results presented in Section 3.1.2 demonstrated that a single fraction can be distinguished by the averaged damping value if the particle size is approximately half the wavelength, but this distinction is more difficult to make for small and large fractions. However, the spectra shown in Section 3.1.1 demonstrated the large difference between the small and large fractions. The following section presents measurements on mixed fractions of quartz. Samples were composed of fractions A and B in different proportions on a percentage weight scale (e.g., 60/40). Figure 15 and Figure 16 show the results for the mixed fractions in a 10 mm sample holder. Multiple measurements were taken at different sample positions, and Figure 15 and Figure 16 show both possible extremes.

The spectra in Figure 15 were selected to demonstrate the influence of the large particle size. Figure 16 shows the lowest influence of the large particle size fraction. This variation has two sources. First, the measurement results highly depend on the local position because of the stochastic arrangement of the particles. Second, the preparation of mixed fractions was extremely complicated because during the infilling of dry material in the sample holder, some separations were visible. For future tests, multiple sample holders will be built, filled with wet mixed material and then oven dried.

In the selected spectra as shown in Figure 15, the small fraction, even at small concentrations, appears to dominate the spectral shape. Conversely, the high damping of the large fraction dominates the averaged damping behavior.

**Figure 17:**
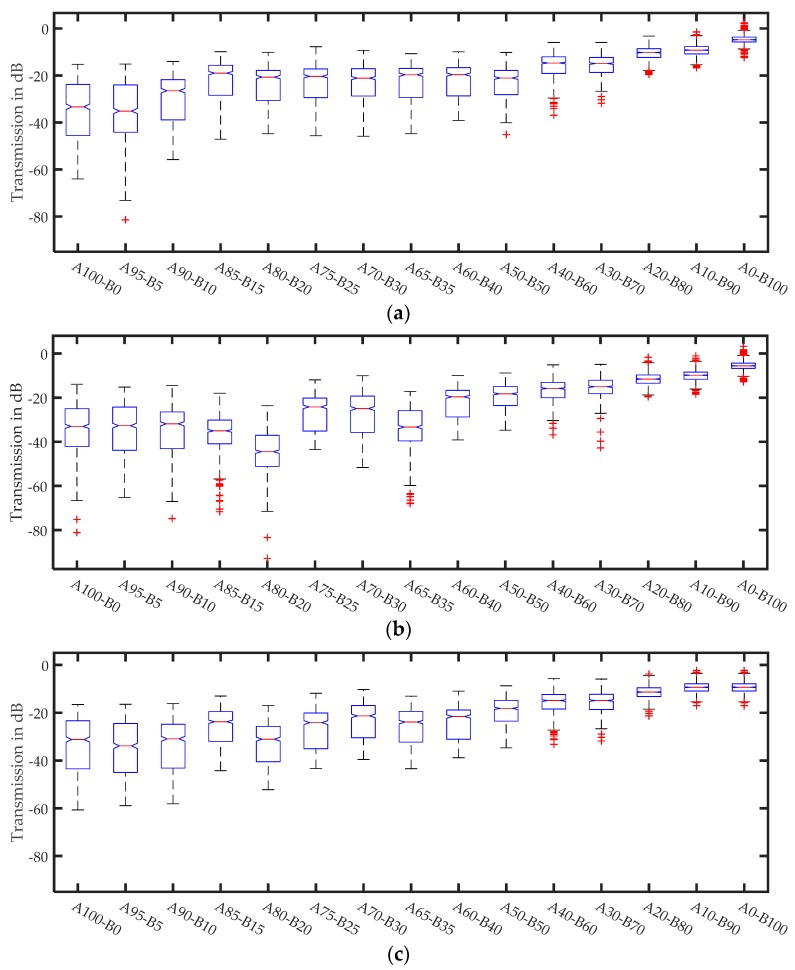
Averaged signal amplitudes for different mixing proportions of fractions A (0.4–0.5 mm) and B (63–100 µm): (**a**) shows the results for the Figure 15 spectra; (**b**) shows the results for the Figure 16 spectra; and (**c**) shows the averaged results of four different sample positions.

**Figure 18:**
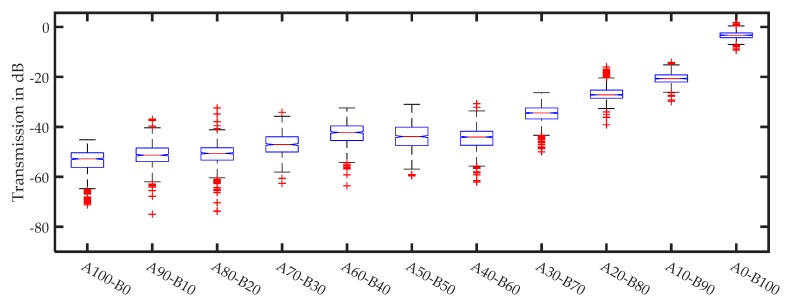
Averaged signal amplitudes for different mixing proportions of fractions A (1.0–1.2 mm) and B (100–210 µm). Averaged results of two different sample positions.

Figure 17 and Figure 18 show the averaged damping results for two mixed fractions. For mixtures with small (<10%) and with high (>70%) proportions of small particles it is evident that damping increased when the proportion of large particles grows. The effect is less prominent with mixtures from 10 to 70% of small particles.

Figure 18 shows the signal damping depending on the mixing proportion. These results demonstrate that the damping of the large particle fraction dominates the behavior up to a proportion of 40/60 (A/B).

### 3.3. Material Content

**Figure 19:**
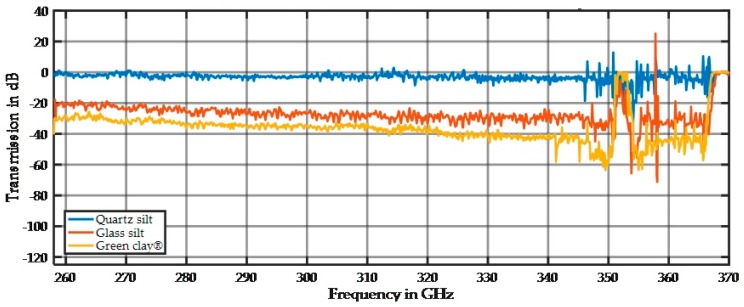
Measurement of quartz silt (45–63 µm), glass silt (<50 µm), and green clay (<2 µm) in the rectangular, 10 mm-thick sample holder. For all measurements, the reference plot obtained for an empty sample holder was subtracted. These plotted results are an average of 10 scans.

Depending on the parent material and the pedogenetic processes, the same particle fraction (e.g., silt) of different soils may have different chemical/mineralogical composition. Thus, with respect to soil texture assessment by THz radiation, it is of interest whether particles of the same size but different chemical composition show similar damping and spectral behavior. We addressed this question by analyzing silt and clay fractions of different mineralogical composition. We selected these fractions because the high surface area of the silt and clay fractions makes these particles the most important for absorption of water and nutrient ions. Figure 19 shows the transmission spectra from three materials with small particles.

**Figure 20:**
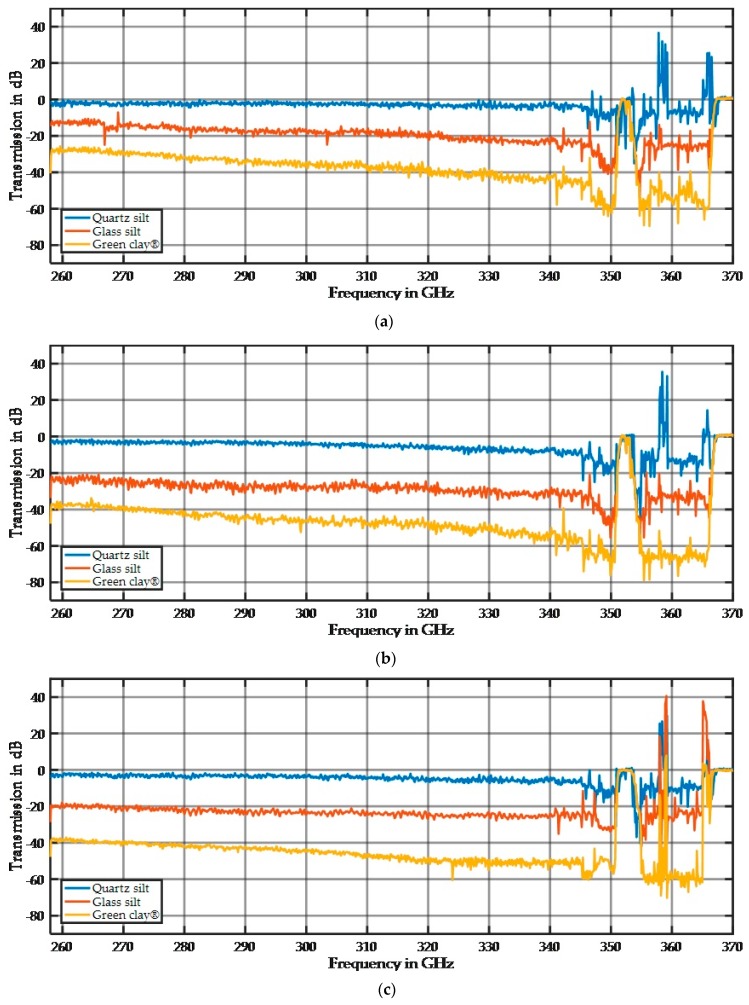
Measurement of quartz silt (45–63 µm), glass silt (<50 µm), and green clay (<2 µm) at three different thicknesses: (**a**) 11 mm; (**b**) 14 mm; and (**c**) 17 mm.

The spectra in Figure 19 were standardized by subtracting the spectrum of the empty sample holder. The small signal damping in Figure 19 shows that these fractions react to THz radiation in a similar way as the bulk material of glass reacts. The damping of a few dB is small and makes these samples transparent in THz investigations. Additionally, the spectral behavior is linear and nearly undisturbed, which makes these materials ideal candidates for doping with ingredients because their spectral behavior does not dominate and can be subtracted from subsequent measurements. In addition, the three different materials have different damping and spectral behaviors and can be precisely distinguished. While the measurements are highly reproducible at a fixed position, the spectrum changes at different positions, especially for the focused beam alignment. This is caused by mechanical variations in the thickness of the sample holder and variations in the particle arrangements and particle size distributions. The dependence of the sample thickness becomes apparent when using the wedge sample holder. Figure 20 shows the spectral response for different sample thicknesses.

The different materials show a difference in their damping (offset) and frequency dependency. While Rayleigh scattering is very small for small particles, the scattering depends on the refraction index for similar particle sizes. The refraction index is a material parameter and thus the material of the sample modifies the absorption behavior. While our samples were made of different materials, they can be distinguished by their signal damping, as shown in Figure 20. Quartz silt is extremely transparent to the considered THz frequencies. Therefore, it is an ideal candidate for mixing with additives in future experiments.

## 4. Discussion

Our results let us conclude that the particle size highly influences the spectral behavior of a transmitted THz signal. Especially if half-wavelength of the THz radiation is similar to the particle size, the averaged damping changes over a range of 40 dB, and the shape of the spectrum changes from a smooth straight line to a highly spiking spectrum with sharp damping peaks. The disadvantage of our measurement setup is that the high-sensitivity range is very small and the signal behavior rapidly saturates on both sides (<0.3 mm and >0.6 mm). Therefore, in future investigations two additional setups for higher and lower frequency ranges are needed to increase the high-sensitivity range.

For particles larger than or equal to the wavelength in size, the spectrum contains many pronounced spikes that are reproducible and cannot be regarded as noise. The positions and arrangement of the spikes change with the sample position. We assume that this results from the stochastic arrangement of the particles. Future work has to investigate if the amount and amplitude of the spikes statistically corresponds to a more precise geometric size (e.g., diameter from 400 µm to 420 µm) or material content. We expect that this resonant scattering also depends on the number of particles in the beam and that more particles allow the spiking move to lower frequencies at large sample thicknesses. Nevertheless, the sharpness of the spikes is impressive and unexpected compared to the simulation results.

On the other hand, the averaged results follow the expected linear behavior for the thickness dependency. Doubling the sample thickness doubles the damping signal on a logarithmic scale.

The situation becomes more complicated for mixed particle size fractions. The sample preparation with dry fractions probably resulted in inhomogeneous samples. It was observed that during the infilling of the sample holders some segregation of the fractions occurred. More sophisticated sample preparation, e.g., by wet mixing and consecutive drying, should be considered in future investigations. However, to overcome the possible segregation effect, the measurements were made at four different heights. For better visibility, we combined the “good” and “worst” results. In the “good” results, the spiking of the large particle fraction was reduced by the small fraction, and the small fraction dominated the spectral behavior for a mixture of 50% large with 50% small (50/50). The damping of the averaged results changed linearly from one fraction to the other. Therefore, the 50/50 mixed fraction cannot be differentiated from the fraction with a size that is intermediate between the small and large fraction sizes based on the damping amplitude. The “good” results show a planar behavior for the averaged damping from 85/15 to 50/50 mixtures, and further investigations must be carried out to understand this behavior. The larger particles dominate the averaged damping signal more in the second mixture up to 40/60. With reduced spiking but higher damping of the 50/50 mixture, the goal of future investigations will be to distinguish a mixed fraction from an intermediate size fraction.

The silt and clay fractions can be easily penetrated by THz radiation because the small particle sizes always cause small Rayleigh scattering. This is important for future investigations because these materials can be used as a matrix combined with ingredients such as organic matter. Additionally, different minerals in the silt and clay sized fractions create different absorption and offset values in the spectra. Future work will investigate distinguishing silt and clay based on the mineralogical composition.

## 5. Conclusions

THz radiation and spectral behavior are highly influenced by the particle size, especially in the range of Mie scattering. Particle sizes in the range of the half-wavelength can be easily distinguished by the large change in the damping level. Ignoring the cost of the setup, the frequency range can be adapted to most soil particle sizes from 100 µm to 2 mm. While the sieved fractions could be distinguished, analyzing an unknown particle size distribution is drastically different. Further research must aim to establish a blind test for the particle size distribution. Additionally, the high transparency of silt and clay materials enables future investigations of the material content and added ingredients. Large particles generate a more complicated signal, but, with respect to the simulation results, also generate many backscatter signals, which could aid in future reflection mode measurements.

## Figures and Tables

**Table 1:** Soil particle size fractions and corresponding THz frequencies.

Clay	Silt	Sand
<2 µm	2–63 µm	63–2000 µm
<150 THz	150–4.76 THz	4.76 THz–150 GHz

**Table 2:** Main chemical components of the different quartz fractions as percentages in proportion of weight.

Element	Fraction				
	<45 µm	45–63 µm	63–100 µm	400–500 µm	1.25–1.40 mm
Si	36.7	38.1	38.4	37.0	37.0
K	3.0	2.5	1.2	0.7	0.5
Al	2.5	2.3	1.4	0.9	0.8
Ti	0.7	0.7	0.6	0.03	0.03
Fe	0.6	0.4	0.2	0.02	0.04

**Table 3:** The main chemical components of the different glass sphere fractions as percentages in proportion of weight.

Element	Fraction			
	<50 µm	100–200 µm	400–600 µm	1.55–1.85 mm
Si	26.6	25.9	27.4	25.8
Ca	5.4	5.1	4.6	4.5
Fe	0.07	0.08	0.04	0.06
Ti	0.02	0.02	0.02	0.02

## Abbreviations

The following abbreviations are used in this manuscript:

Vis-NIR	visible to near-infrared
2D	two-dimensional
HDPE	high-density polyethylene
THz	Terahertz
GHz	Gigahertz
pH	Potential hydrogen
XRF	Roentgen radiation fluorescence or X-ray fluorescence
BWO	Backward wave oscillator (tube)
CW	Continuous wave
HM	Harmonic mixer
